# Optimization of Assembly Pipeline may Improve the Sequence of the Chloroplast Genome in *Quercus spinosa*

**DOI:** 10.1038/s41598-018-27298-0

**Published:** 2018-06-11

**Authors:** Xiangzhou Zhang, Yong Hu, Mei Liu, Tiange Lang

**Affiliations:** Big Data Decision Institute, Jinan University, Tianhe, Guangzhou P.R. China

## Abstract

Obtaining chloroplast (cp) genome sequence is necessary for studying physiological roles in plants. However, it is difficult to use traditional sequencing methods to get cp genome sequences because of the complex procedures of preparing templates. With the advent of next-generation sequencing technology, massive genome sequences can be produced. Thus, a good pipeline to assemble next-generation sequence reads with optimized k-mer length is essential to get whole cp genome sequences. Moreover, adjustment of other parameters is also very important, especially for the assembly of the cp genome. In this study, we developed a pipeline to generate the cp genome for *Quercus spinosa*. When *Quercus rubra* was used as a reference, we achieved coverage of 97.75% after optimizing k-mer length as well as other parameters. The efficiency of the pipeline makes it a useful method for cp genome construction in plants. It also provides great perspective on the analysis of cp genome characteristics and evolution.

## Introduction

Chloroplast (cp) genome resources, both in model organisms and non-model organisms, have been extensively used in molecular ecology and evolution studies^1,2^. However, there are still many species for which the cp genomes have not been fully sequenced^3^. Since the release of the first cp genome sequence, there have been numerous improvements in chloroplast DNA extraction methods^4–6^, and the field is now rich with many sequence assembly tools^7–9^. Compared with cp isolation and DNA extraction, the process of cp genome assembly from a large amount of short-read sequencing data is more difficult^10^.

The assembly of cp genomes continues to be a fundamental part of the analysis of high-throughput sequencing data^10,11^. In recent studies, two strategies have been used in cp genome assembly: one is a reference-based assembly method, and the other is a *de novo* assembly method^11–13^. For organisms without a reference cp genome, researchers are inclined to use *de novo* assembly strategies, mostly based on *de bruijn* graph methods, which rely significantly on a parameter k (defined as the length of k-mer)^14^. The choice of tools and parameters in the analysis process represents a trade-off among several competing effects. For example, methods choosing small k-mers have the advantages of allowing assembly of sequences with low coverage depth and removing sequencing errors, while those choosing large k-mers are efficient for assembling repeat regions but give a higher chance of introducing errors^7,14^.

Recently, several researchers have provided solutions for problems in the *de novo* assembly process^11,14,15^. However, there has been no report on how to choose a suitable pipeline or a best specific parameter in the cp genome assembly process. We designed this study to evaluate the effect of different values of several parameters in the quality control and assembly steps. Our results indicated that: (1) the minimum read length had little effect on the result of assembly; and (2) when k-mer size was set to 81 with a sequence read length of 100 base pairs, the assembly of the cp genome of *Quercus spinosa* was most complete. Therefore, this study implied that optimization of the assembly pipeline could improve the cp genome.

## Materials and Methods

### Next-generation sequencing data

The sequencing reads of *Q*. *spinosa* plants used to build the cp genome of *Q*. *spinosa* were downloaded from the NCBI SRA database under the accession number SRP061187.

### Quality control methods

The raw data from the Illumina Hiseq2000 were trimmed with two programs for performing quality control written in the Practical Extraction and Report Language (PERL). The first program was used to remove nucleotides with a Phred score lower than 13, which means the probability of a nucleotide to be erroneous is 0.05 (Supplementary Script S1). The second program was used to delete fastq reads created by the first program with length less than a certain value (Supplementary Script S2). For that certain value, we tested 17, 21 and 25.

### Sequence assembly

We used SOAPdenovo2 (http://soap.genomics.org.cn/soapdenovo.html) to align the trimmed Illumina fastq reads to obtain contigs^16,17^. This software was particularly designed to assemble Illumina GA short reads. To get the best alignment, we tested different possible k-mer lengths, which represented odd numbers starting from 17 and ending with 99. For other parameters, we employed 500 as average insert size, 3 as cutoff value of pair number for a reliable connection between two contigs of pre-scaffolds, and 32 as minimum alignment length between a read and contig required for a reliable read location.

### Length control

Contigs shorter than 150 base pairs were removed because the length of the raw read was 100 base pairs. Since we used three numbers (17, 21 and 25) as the minimum read length and 42 numbers (odd numbers from 17 to 99) as k-mer length, we got 126 contigs in total.

### Similarity search

BLAST (http://blast.ncbi.nlm.nih.gov/Blast.cgi) was used for similarity search. The query sequences were the assembled contigs with different k-mers after length control with different minimum lengths. The database searched was the complete cp genome of *Quercus rubra*, which was downloaded from the NCBI database (http://www.ncbi.nlm.nih.gov/genome/).

## Results and Discussion

### Chloroplast genomic contigs could be constructed from Illumina cp genomic sequence data

The Illumina Hiseq2000 cp genomic sequence data in fastq format for *Q*. *Spinosa* was 1.4 Gigabytes, and it had 561,845,830 base pairs. We used quality control methods in dealing with the Illumina cp sequence data. These methods improved the quality of the sequence data by a small extent, since the high-quality sequence data could be reduced to 1.3 Gigabytes. We used SOAPdenovo2 to align the trimmed Illumina fastq reads for generating contigs.

**Figure 1:**
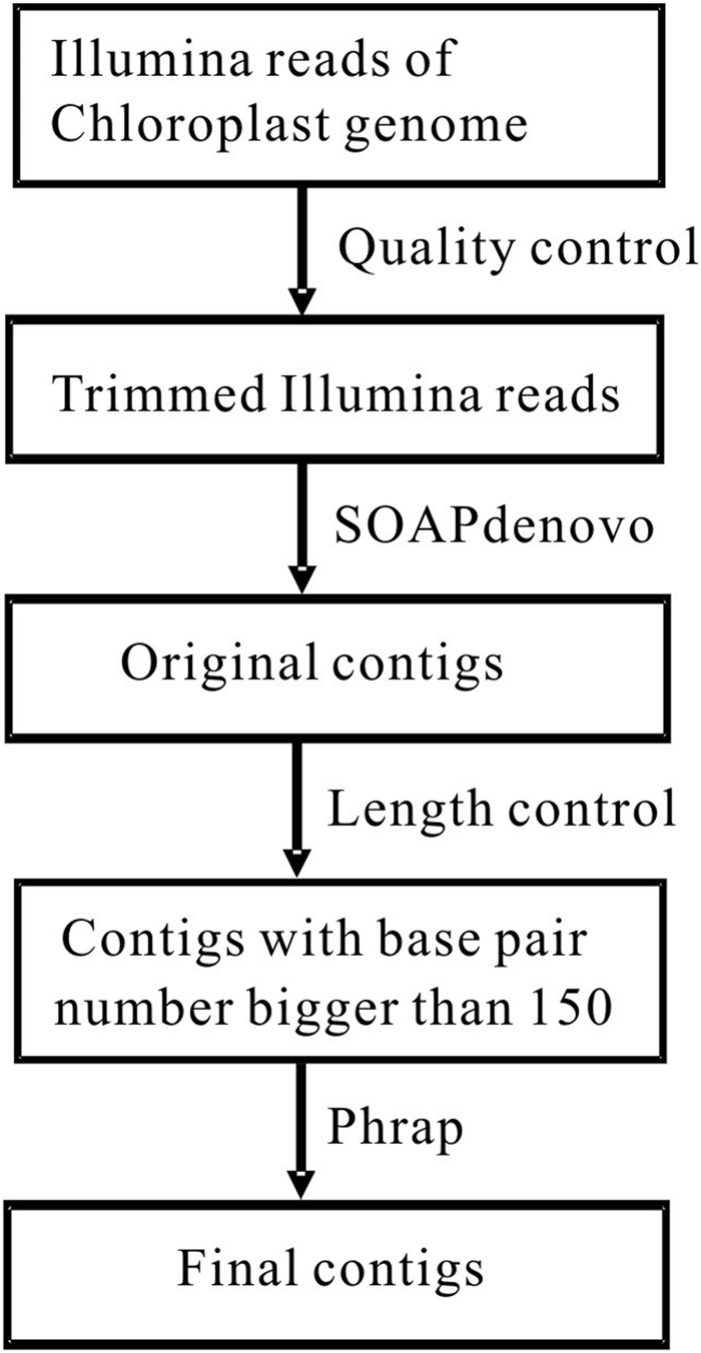
Proposed programming pipeline for predicting cp genomic contigs of *Q*. *spinosa*. Illumina reads were first trimmed with quality control methods. The assembly software SOAPdenovo2 was then used separately to obtain original contigs. Length control methods were next used to select contigs larger than 150 base pairs. Afterwards, the assembly software Phrap was used to obtain final contigs.

For the similarity search, it was most appropriate to use the contigs that had a length >150 base pairs as the input for BLAST as the length of the best HSP (High Score Pair) from the BLAST results was about 150. Therefore, we picked the aligned contigs that had lengths greater than 150 base pairs from the results of SOAPdenovo2. We used Phrap on those contigs to remove the sequence redundancy and improve contig alignment (Fig. 1).

### Choice of minimum read length did not affect total HSP length

Nucleotides in sequence reads with Phred score less than 13 should be removed. However, the deletion of only one nucleotide would cause a frame shift. Thus, we removed the whole DNA segment from the nucleotide with the low Phred score to near the end of the read. Therefore, read lengths were reduced.

**Figure 2:**
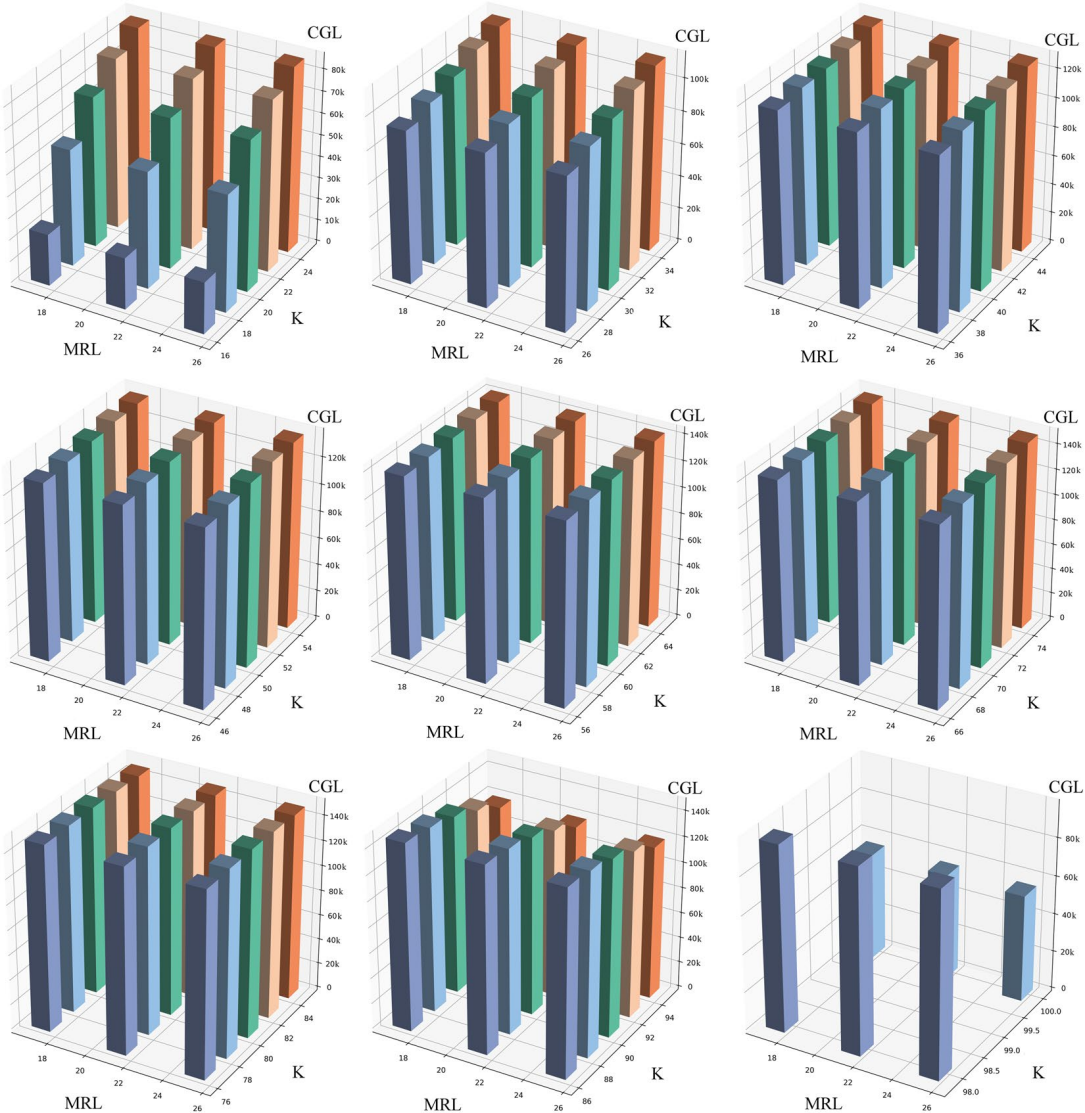
Lengths of the constructed cp genome of *Q*. *spinosa* as shown by bars. The k-values of k-mers used in the assembly step were from 17 to 99 (odd numbers), with different colors of bars in each box. The minimum read lengths used in the length control step were 17, 21 and 25 base pairs, respectively. The result of the constructed cp genome of *Q*. *spinosa* could be affected obviously by the k-values of k-mers used in the assembly step, but not the minimum read lengths used in the length control step. CGL = constructed chloroplast genome length of *Q*. *spinose*. MRL = minimum read length. K = k-value.

We tested the effect of choosing different minimum read lengths for assembly. With different values of minimum read length and k-mer length, different genomic contigs of cp could be built. Using the cp genome sequence of *Q*. *rubra* as the reference sequence, the cp genome of *Q*. *spinosa* could be constructed. The constructed *Q*. *spinosa* cp genomes were almost the same with different minimum read lengths, but they varied a lot with different k-values of the k-mers (Fig. 2).

### Selection of the best k-mer in the assembly step greatly affects the construction of the cp genome

The cp genome of *Q*. *spinosa* was constructed based on the result of the BLAST search, using the assembled contigs from the Illumina reads of the cp genome of *Q*. *spinosa* as queries and the whole genomic sequence of the *Q*. *rubra* cp as the database. The total lengths of all HSPs from the BLAST results were calculated for each BLAST process. Only the best HSP was kept for each query sequence, and this step could remove the redundancy of the HSPs. The lengths of HSPs of the reference sequence (*Q*. *rubra* cp genome) with *Q*. *spinosa* cp contigs assembled with different k-mers (k was an odd number from 17 to 99) were calculated. We set the minimum k-mer length as 17 since from 17 upwards, the number of theoretical k-mers (the 17th power of 4) was much bigger than the number of real k-mers. We set the maximum k-mer length as 99 since the sequence read length was 100 and we could not get k-mers that had a length greater than 100. We only chose odd numbers as k-mer lengths since some of the k-mers with lengths being even numbers would have had the same complementary sequence, and thus these k-mers could cause statistical bias.

**Figure 3:**
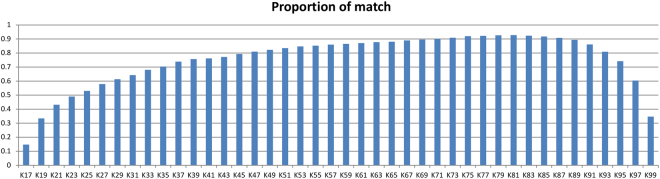
Proportions of the *Q*. *spinosa* cp genome constructed with different k-values of the k-mers used in the assembly step before filling in the gaps. The best k-value was 81, with a proportion of 92%.

For the best HSPs, we only kept those with identity greater than 85% and length greater than 150 base pairs. The reason for choosing 85% identity was that *Q*. *spinosa* and *Q*. *rubra* have sequences that are 85% identical. The reason for choosing 150 as the HSP length cutoff was that the minimum building segment length of the *Q*. *spinosa* cp genome should be around 150. With those criteria, the cp genome sequences of *Q*. *spinosa* could be built using the *Q*. *rubra* cp genome as a reference. The total number of base pairs of the *Q*. *spinosa* cp genome constructed with different assembled k-mers is shown in Supplementary Table 1. The best k-value of k-mer was 81, giving a proportion of 92.81% of the total cp genome (Fig. 3). The other 7.19% was gaps, which could be filled in with Illumina reads.

**Figure 4:**
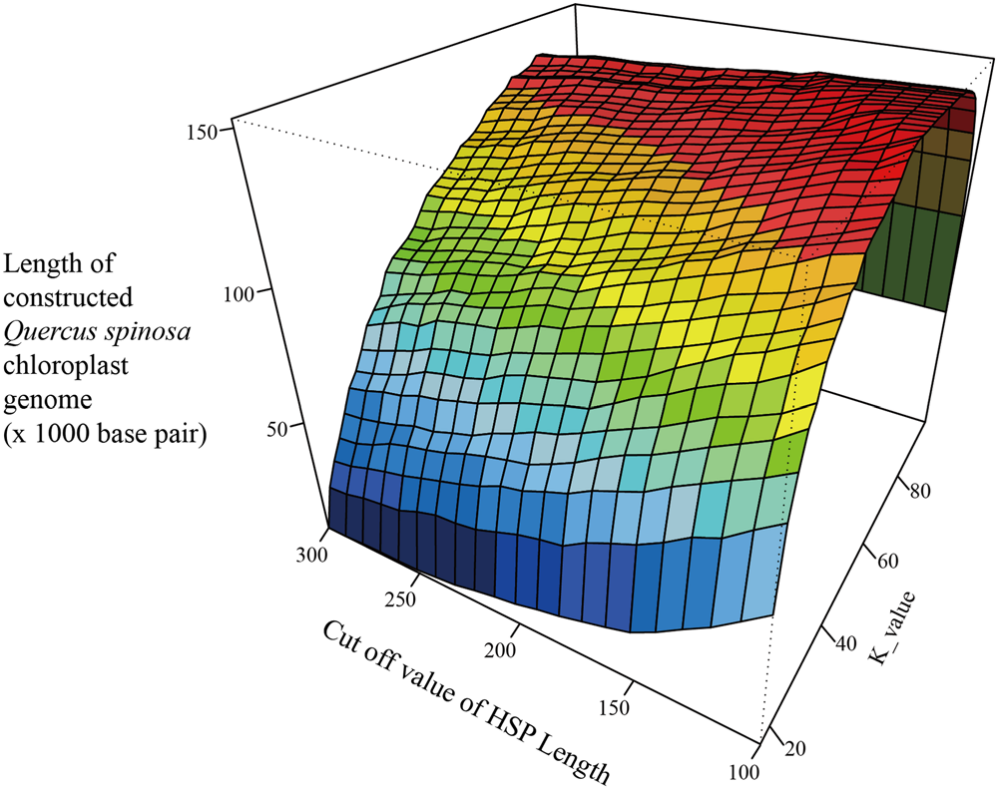
The curve surface of lengths of the constructed **Q. spinosa** cp genome with different k-values of the k-mers used in the assembly step, odd numbers from 17 to 99, and different cutoff values of HSP lengths used in the similarity search step. The blue, green, yellow and red colors represent k-values as 17–39, 41–59, 61–79 and 81–99, respectively. The HSP lengths were from 100 to 300 in multiples of 10. The important affecter is the k-value of the k-mer used in the assembly step.

We also tested the different cutoff values of HSP length from 150 to 300 base pairs, and got a curved surface that had a peak line almost horizontal, and the k-value of the peak was about 81 (Fig. 4). Therefore, the crucial parameter of the cp genome assembly pipeline was the k-value of the k-mer that was used in the contig assembly step.

### Construction of the cp genome of *Q*. *spinosa*

With the most optimal k-mer and the most optimal minimum read length, the cp genome Illumina reads of *Q*. *spinosa* could be assembled into contigs. The assembly results are shown in Supplementary Table 2. Similarity searches of the assembled contigs were performed with the *Q*. *rubra* cp genome as a reference genome. Contigs with HSP values showing identities greater than 80%, e-values less than 1e-30 and HSP lengths of more than 150 were selected for the construction of the cp genome of *Q*. *spinosa*.

We aligned the Illumina reads back to the assembled contigs that were selected for building the cp genome of *Q*. *spinosa*, and 988,357 pair ends could be aligned back. The total number of pair ends of the cp genome of *Q*. *spinosa* was 2,624,649, thus the percentage of reads that could be used was 37.65%. The constructed length of the *Q*. *spinosa* cp genome after our pipeline was 149,703 base pairs. After filling in the gaps using the Illumina reads, the proportion of the *Q*. *spinosa* cp genome constructed reached 97.75%.

## Electronic supplementary material

Supplementary information

## References

[1] Du FK, Petit RJ, Liu JQ (2009). More introgression with less gene flow: chloroplast vs. mitochondrial DNA in the Picea asperata complex in China, and comparison with other Conifers. Mol Ecol.

[2] Xu T (2010). Phylogeography and allopatric divergence of cypress species (Cupressus L.) in the Qinghai-Tibetan Plateau and adjacent regions. BMC Evol Biol.

[3] Aslan CE, Zavaleta ES, Tershy B, Croll D (2013). Mutualism Disruption Threatens Global Plant Biodiversity: A Systematic Review. Plos One.

[4] Atherton RA (2010). Whole genome sequencing of enriched chloroplast DNA using the Illumina GAII platform. Plant Methods.

[5] Huang DI, Hefer CA, Kolosova N, Douglas CJ, Cronk QC (2014). Whole plastome sequencing reveals deep plastid divergence and cytonuclear discordance between closely related balsam poplars, Populus balsamifera and P. trichocarpa (Salicaceae). New Phytol.

[6] Nock CJ (2011). Chloroplast genome sequences from total DNA for plant identification. Plant Biotechnol J.

[7] Luo R (2012). SOAPdenovo2: an empirically improved memory-efficient short-read *de novo* assembler. Gigascience.

[8] Simpson JT (2009). ABySS: a parallel assembler for short read sequence data. Genome Res.

[9] Zerbino DR, Birney E (2008). Velvet: algorithms for *de novo* short read assembly using de Bruijn graphs. Genome Res.

[10] Miller JR, Koren S, Sutton G (2010). Assembly algorithms for next-generation sequencing data. Genomics.

[11] Chikhi R, Medvedev P (2014). Informed and automated k-mer size selection for genome assembly. Bioinformatics.

[12] Cronn R (2008). Multiplex sequencing of plant chloroplast genomes using Solexa sequencing-by-synthesis technology. Nucleic Acids Res.

[13] Ferrarini M (2013). An evaluation of the PacBio RS platform for sequencing and *de novo* assembly of a chloroplast genome. BMC Genomics.

[14] Zhao QY (2011). Optimizing *de novo* transcriptome assembly from short-read RNA-Seq data: a comparative study. BMC Bioinformatics.

[15] Surget-Groba Y, Montoya-Burgos JI (2010). Optimization of *de novo* transcriptome assembly from next-generation sequencing data. Genome Res.

[16] Li R (2009). SOAP2: an improved ultrafast tool for short read alignment. Bioinformatics.

[17] Li R (2010). *de novo* assembly of human genomes with massively parallel short read sequencing. Genome Res.

