# Supplementary material for: Optimization of Assembly Pipeline may Improve the Sequence of the Chloroplast Genome in Quercus spinosa
Source: Sci Rep. 2018 Jun 11;8:8906. doi: 10.1038/s41598-018-27298-0 (PMC5995970; doi:10.1038/s41598-018-27298-0)
Supplement: Supplementary file 1 — Supplementary information [file 41598_2018_27298_MOESM1_ESM.pdf]

Supplementary Scripts and Tables to

Optimization of Assembly Pipeline may Improve the Sequence of the Chloroplast Genome in *Quercus spinosa*

Xiangzhou Zhang[1], Yong Hu[1], Mei Liu[1] and Tiange Lang[1,*]

Script S1 - S2

Script S1. FastqTrim_Quality.pl: This program was used to remove the nucleotides which have Phred score lower than a specific value.

Script S2. fastqTrim_Length.pl: This program was used to delete the fastq reads which have length less than a specific value as well as to erase the "orphanage" reads (single reads without pair).

Table S1 – S2

Table S1. The numbers of base pairs of the constructed *Quercus spinosa* cp genome with different assembled k-mers. The longest genome was constructed with k-value of k-mer 81.

Table S2. The assembly results of *Quercus spinosa* chloroplast genome contigs with the most optimal K-mer and the most optimal minimum read length.

Script S1

:::::::::::::
fastqTrim_Quality.pl: This program was used to remove the nucleotides which have Phred score lower than a specific value.
:::::::::::::

```
#!/usr/bin/env perl

use strict;
use warnings;
use Getopt::Long;
use File::Spec;
my $usage = "
$0 input_files [-p|probcutoff 0.05] [-h|phredcutoff 13] [-b|bwa] [-d|directory path] [-sanger
-solexa -illumina] [-454]\n
-p|probcutoff probability value (between 0 and 1) at which base-calling error is considered too
high (default; p = 0.05) *or*
-h|phredcutoff    Phred score (between 0 and 40) at which base-calling error is considered too
high
-b|bwa                use BWA trimming algorithm
-d|directory        path to directory where output files are saved
-sanger               Sanger format (bypasses automatic format detection)
-solexa               Solexa format (bypasses automatic format detection)
-illumina            Illumina format (bypasses automatic format detection)
-454                    set flag if trimming Roche 454 data (experimental feature)
\n";
if( !$ARGV[0] ){ die "$usage"; }
my $prob_cutoff;
my $phrd_cutoff;
my $ascii_cutoff;
my $automatic_detection_lines = 10000;
my $sanger;
my $solexa;
my $illumina;
my $format;
my $user_defined;
my $bwa;
my $directory;
my $roche;
my $poor_quality_char = "B";
GetOptions(
	"p|probcutoff=f"    => \$prob_cutoff,
	"h|phredcutoff=f" => \$phrd_cutoff,
```

```
	"b|bwa"			=> \$bwa,
	"d|directory=s"	=> \$directory,
	"sanger"		=> \$sanger,
	"solexa"		=> \$solexa,
	"illumina"		=> \$illumina,
	"454"			=> \$roche
);
if( ($sanger && $solexa) || ($sanger && $illumina) || ($solexa && $illumina) ){
	die "error: please select only one of -sanger, -solexa or -illumina\n";
}
if( $sanger || $solexa || $illumina ){
	$user_defined = 1;
}
if( $sanger ){
	$format = "sanger";
}elsif( $solexa ){
	$format = "solexa";
}elsif( $illumina ){
	$format = "illumina";
}
if( $roche ){
	$format = "sanger";
}
my @files = @ARGV;
if( !$files[0] ){ die "$usage"; }
if( !defined( $prob_cutoff ) && !defined( $phrd_cutoff ) ){
	$prob_cutoff = 0.05;
	print STDOUT "Info: Using default quality cutoff of P = $prob_cutoff (change with -p or -h
flag)\n";
}elsif( defined( $prob_cutoff ) && defined( $phrd_cutoff ) ){
	die "Error: Please enter either a probability or a Phred quality cutoff value, not both";
}elsif( defined( $prob_cutoff ) && ( $prob_cutoff < 0 || $prob_cutoff > 1 ) ){
	die "Error: P quality cutoff must be between 0 and 1";
}elsif( defined( $phrd_cutoff ) && $phrd_cutoff < 0 ){
	die "Error: Phred quality cutoff must be greater than or equal to 0";
}
if( !`which R 2> err.log` ){
	print STDERR "Warning: Subsidiary program R not found. Histogram will not be
produced.\n";
}
`rm err.log`;
foreach my $input_file ( @files ){
	open( INPUT, "<$input_file" ) or die "Error: Failure opening $input_file for reading: $!\n";
	my @filepath = split( /\//, $input_file );
```

```
	my $filename = $filepath[$#filepath];
	if( !$user_defined ){
		$format = "";
	}
	if( !$format ){

		$format = &get_format(*INPUT, $automatic_detection_lines);
		if( !$format ){
			die "Error: File format cannot be determined\n";
		}
	}
	my %dict_q_to_Q;
	%dict_q_to_Q=&q_to_Q();
	if( $format eq "sanger" ){
		$poor_quality_char = "!";
	}elsif( $format eq "solexa" ){
		$poor_quality_char = ";";
	}elsif( $format eq "illumina" ){
		$poor_quality_char = "@";
	}
	if( $roche ){
		print STDOUT "User defined format: Roche 454, Sanger FASTQ format\n";
	}elsif( $format eq "sanger" ){
		if( $user_defined ){
			print STDOUT "User defined format: Sanger FASTQ format\n";
		}else{
			print STDOUT "Automatic format detection: Sanger FASTQ format\n";
		}
	}elsif( $format eq "solexa" ){
		if( $user_defined ){
			print STDOUT "User defined format: Solexa FASTQ format, Illumina pipeline 1.2 or
less\n";
		}else{
			print STDOUT "Automatic format detection: Solexa FASTQ format, Illumina
pipeline 1.2 or less\n";
		}
	}elsif( $format eq "illumina" ){
		if( $user_defined ){
			print STDOUT "User defined format: Illumina FASTQ format, Illumina pipeline
1.3+\n";
		}else{
			print STDOUT "Automatic format detection: Illumina FASTQ format, Illumina
pipeline 1.3+\n";
		}
```

```
}
if( defined( $phrd_cutoff ) ){
	$ascii_cutoff = &Q_to_q( $phrd_cutoff );
	$prob_cutoff = sprintf("%.5f", &Q_to_p( $phrd_cutoff ));
}else{
	$ascii_cutoff = &Q_to_q( &p_to_Q( $prob_cutoff ) );
}
my $threshold = 0;
if( $bwa ){

	if( defined( $phrd_cutoff ) ){
		$threshold = $phrd_cutoff;
	}else{
		$threshold = &p_to_Q( $prob_cutoff );
	}
}
my $output_file;
if ( $directory ){
	# remove any trailing '/'
	$directory =~ s/\/\z//;
	my $file_name = $filename . ".trimmed";
	$output_file = File::Spec->catpath( undef, $directory, $file_name );
}else{
	$output_file = $filename . ".trimmed";
}

if( -e $output_file ){
	die "Error: Output file $output_file already exists: $!\n";
}
open( OUTPUT, ">$output_file" )
			or die "Error: Failure opening $output_file for writing: $!\n";
my @segment_hist;
my %hash=();
my $segment_sum	= 0;
my $segment_count = 0;
my $original_length;
my $seq_count = 0;
while( <INPUT> ){
	my $ID1 = $_;
	if( substr( $ID1, 0 , 1) ne "@" ){
		die "Error: Input file not in correct FASTQ format at seq ID $ID1\n";
	}
	chomp( my $seq_string = <INPUT> );
	my $ID2 = <INPUT>;
```

```
if( substr( $ID2, 0 , 1) ne "+" ){
	die "Error: Input file not in correct FASTQ format at qual ID $ID2\n";
}
chomp( my $quality_string = <INPUT> );
$original_length    = length $seq_string;
my $cutoff_hit              =    0;
my $best_start_index =    0;
my $best_length            =    0;
my $current_start        =    0;
my $bad_first               =    0;
if( $bwa ){
	my @qual = split(//, $quality_string );
	for( my $i = 0; $i < scalar @qual; $i++ ){

		$qual[$i] = $dict_q_to_Q{$qual[$i]};
	}
	if( !$qual[0] ){
		$bad_first = 1;
		$best_length = 0;

	}elsif( $qual[0] < $threshold ){
		$bad_first = 1;
		$best_length = &bwa_trim( $threshold, \@qual );

	}else{
		$best_length = &bwa_trim( $threshold, \@qual );
	}
}else{
	for( my $i = 0; $i < $original_length; $i++ ){
		if( substr($quality_string, $i, 1) le $ascii_cutoff ){
			$cutoff_hit = 1;
			my $current_segment_length = $i - $current_start;
			if( $current_segment_length > $best_length ){
				$best_length            = $current_segment_length;
				$best_start_index = $current_start;
			}
			$current_start = $i + 1;
		}elsif( $i == $original_length - 1){
			my $current_segment_length = ($i + 1) - $current_start;
			if( $current_segment_length > $best_length ){
				$best_length = $current_segment_length;
				$best_start_index = $current_start;
			}
		}
```

```
			}
			if( !$cutoff_hit ){
				$best_length = $original_length;
			}
		}
		if( !defined($segment_hist[ $best_length ] ) ){
			$segment_hist[ $best_length ] = 0;
		}
		$segment_hist[ $best_length ]++;
		$segment_sum += $best_length;
		$segment_count++;
		if (exists $hash{$best_length}) {
				$hash{$best_length}+=1;
					}
		else{
				$hash{$best_length}=1
					}
		if( $bwa ){
			if( $best_length <= 1 && $bad_first ) {
				$seq_string = "N";
				$quality_string = $poor_quality_char;
			}else{
				$seq_string = substr($seq_string, 0, $best_length);
				$quality_string = substr($quality_string, 0, $best_length);
			}
		}else{
			if ($best_length <= 0) {
				$seq_string = "N";
				$quality_string = $poor_quality_char;
			} else {
				$seq_string = substr($seq_string, $best_start_index, $best_length);
				$quality_string = substr($quality_string, $best_start_index, $best_length);
			}
		}
		print OUTPUT $ID1, $seq_string, "\n", $ID2, $quality_string, "\n";

	}
	my $segment_mean = sprintf( "%.1f", $segment_sum / $segment_count );
	my $halfway_index = $segment_count / 2;
	my $current_sum	= 0;
	my $current_index = 0;
	my $median_index1;
	my $median_index2;
	while( !defined( $median_index1 ) || !defined( $median_index2 ) ){
```

```
			if( defined( $segment_hist[ $current_index ] ) ){

				$current_sum += $segment_hist[ $current_index ];
			}
			if( $current_sum > $halfway_index ){
				if( !defined( $median_index1 ) ){
					$median_index1 = $current_index;
				}
				if( !defined( $median_index2 ) ){
					$median_index2 = $current_index;
				}
			}elsif( $current_sum == $halfway_index	&& !defined( $median_index1 ) ){
				$median_index1 = $current_index;
			}
			$current_index++;
		}
		$current_index--;
		my $segment_median;
		if( $segment_count % 2 == 1){
			$segment_median = $median_index1;
		}else{
			$segment_median = sprintf( "%.0f", ( ( $median_index1 + $median_index2 ) / 2 ) );
		}
		print STDOUT "Info: $output_file: mean segment length = $segment_mean, median
segment length = $segment_median\n";
		close INPUT or die "Error: Cannot close $input_file: $!";
		close OUTPUT or die "Error: Cannot close $output_file; $!";
		my $segments_filename;
		if ( $directory ){
			$segments_filename="$directory/$filename.trimmed_segments";
						}
		else{
			$segments_filename="$filename.trimmed_segments";
					}
		open(SEGMENTS, ">$segments_filename");
		print SEGMENTS "Read_length\tProportion_of_reads\n";
		my $i;
		for ($i=0;$i <= $original_length; $i++){
			if (exists $hash{$i}){
				my $percentage=$hash{$i}/$segment_count;
				print SEGMENTS "$i\t$percentage\n";		}
			else{print SEGMENTS "$i\t0\n";
							}
							}
```

```
    close SEGMENTS or die "Error: Cannot close $segments_filename; $!";
}
```

Script S2

:::::::::::::

fastqTrim_Length.pl: This program was used to delete the fastq reads which have length less than a specific value as well as to erase the "orphanage" reads (single reads without pair).

:::::::::::::

```
#!/usr/bin/env perl

use strict;
use warnings;
use Getopt::Long;
use File::Spec;
my $length = 25;
my $paired = 0;
my $directory;
my $usage = "
$0 one single-end or two paired-end FASTQ files [-l|length 25] [-d|directory path]\n
-l|length         length cutoff [defaults to 25 nucleotides]
-d|directory      path to directory where output files are saved
\n";
GetOptions(
	"l|length=i"	=> \$length,
	"d|directory=s"	=> \$directory
);
my @files = @ARGV;
if( !$files[0] || length(@files) > 2 ){
	die $usage;
}
if( scalar(@files) == 2 ){
	$paired = 1;
}
unless( -e $files[0] ){
	die "error: file $files[0] does not exist\n";
}
open( FIRST, "<$files[0]" )
	or die "error: failure opening $files[0] for reading: $!\n";
if( $paired ){
	unless( -e $files[1] ){
		die "error: file $files[1] does not exist\n";
	}
	open( SECOND, "<$files[1]" )
		or die "error: failure opening $files[1] for reading: $!\n";
}
```

```
my $first_line;
my $second_line;
$first_line = <FIRST>;
if( substr($first_line, 0, 1) ne "@" ){
	die "error: $files[0] does not appear to be in FASTQ format\n";
}
if( $paired ){
	$second_line = <SECOND>;
	if( substr($second_line, 0, 1) ne "@" ){
		die "error: $files[1] does not appear to be in FASTQ format\n";
	}
}
my $first_id;
my $second_id;
if( $paired ){
	if( $first_line !~ /\S+\s\S+/ ){
		if( $first_line =~ /(\S*)\/\S*/ ){
			$first_id = $1;
		}else{
			$first_id = $first_line;
		}
	}elsif( $first_line =~ /\S+\s\S+/ ){
		my @first_line_elements = split( /\s+/, $first_line );
		pop @first_line_elements;
		$first_id = join( " ", @first_line_elements );
	}else{
		$first_id = $first_line;
	}
	if( $second_line !~ /\S+\s\S+/ ){
		if( $second_line =~ /(\S*)\/\S*/ ){
			$second_id = $1;
		}else{
			$second_id = $second_line;
		}
	}elsif( $second_line =~ /\S+\s\S+/ ){
		my @second_line_elements = split( /\s+/, $second_line );
		pop @second_line_elements;
		$second_id = join( " ", @second_line_elements );
	}else{
		$second_id = $second_line;
	}
	if( $first_id ne $second_id ){
		die "error: files $files[0] and $files[1] do not seem to be paired\n";
	}
```

```
}
if( $paired ){
	my $first_line_counter = 0;
	my $second_line_counter = 0;
	$first_line_counter++ while <FIRST>;
	$second_line_counter++ while <SECOND>;
	if( $first_line_counter != $second_line_counter ){
		die "error: files $files[0] and $files[1] appear to be different lengths\n";
	}
}
seek(FIRST, 0, 0);
if( $paired ){
	seek(SECOND, 0, 0);
}
my $single_file;
if ( $directory ){
	$directory =~ s/\/\z//;
	my @file_ending_elements = split(/\//, $files[0]);
	my $item = scalar @file_ending_elements - 1;
	my $file_name = $file_ending_elements[$item] . ".single";
	$single_file = File::Spec->catpath( undef, $directory, $file_name );
}else{
	$single_file = $files[0] . ".single";
}
if( -e $single_file ){
	die "error: file $single_file already exists\n";
}
open( SINGLE, ">$single_file" )
	or die "error: failure opening $single_file for writing: $!\n";
my $discard_file;
if ( $directory ){
	$directory =~ s/\/\z//;
	my @file_ending_elements = split(/\//, $files[0]);
	my $item = scalar @file_ending_elements - 1;
	my $file_name = $file_ending_elements[$item] . ".discard";
	$discard_file = File::Spec->catpath( undef, $directory, $file_name );
}else{
	$discard_file = $files[0] . ".discard";
}
if( -e $discard_file ){
	die "error: file $discard_file already exists\n";
}
open( DISCARD, ">$discard_file" )
	or die "error: failure opening $discard_file for writing: $!\n";
```

```
my $paired_file1;
my $paired_file2;
if( $paired ){
	if ( $directory ){
		$directory =~ s/\/\z//;
		my @file_ending_elements = split(/\//, $files[0]);
		my $item = scalar @file_ending_elements - 1;
		my $file_name = $file_ending_elements[$item] . ".paired1";
		$paired_file1 = File::Spec->catpath( undef, $directory, $file_name );
	}else{
		$paired_file1 = $files[0] . ".paired1";
	}
	if( -e $paired_file1 ){
		die "error: file $paired_file1 already exists\n";
	}
	open( PAIRED1, ">$paired_file1" )
		or die "error: failure opening $paired_file1 for writing: $!\n";
	if ( $directory ){
		$directory =~ s/\/\z//;
		my @file_ending_elements = split(/\//, $files[0]);
		my $item = scalar @file_ending_elements - 1;
		my $file_name = $file_ending_elements[$item] . ".paired2";
		$paired_file2 = File::Spec->catpath( undef, $directory, $file_name );
	}else{
		$paired_file2 = $files[0] . ".paired2";
	}
	if( -e $paired_file2 ){
		die "error: file $paired_file2 already exists\n";
	}
	open( PAIRED2, ">$paired_file2" )
		or die "error: failure opening $paired_file2 for writing: $!\n";
}
my $count_p1=0;
my $count_p2=0;
my $count_d=0;
my $count_s=0;
until( eof(FIRST) ){
	chomp( my $first_header_line1 = <FIRST> );
	chomp( my $first_sequence_line = <FIRST> );
	chomp( my $first_header_line2 = <FIRST> );
	chomp( my $first_quality_line = <FIRST> );
	my $second_header_line1;
	my $second_sequence_line;
	my $second_header_line2;
```

```
my $second_quality_line;
if( $paired ){
	chomp( $second_header_line1 = <SECOND> );
	chomp( $second_sequence_line = <SECOND> );
	chomp( $second_header_line2 = <SECOND> );
	chomp( $second_quality_line = <SECOND> );
}
if( $paired ){
	my $first_header_id;
	my $second_header_id;
	if( $first_header_line1 !~ /\S+\s\S+/ ){
		if( $first_header_line1 =~ /(\S*)\/\S*/ ){
			$first_header_id = $1;
		}else{
			$first_header_id = $first_header_line1;
		}
	}elsif( $first_header_line1 =~ /\S+\s\S+/ ){
		my @first_header_line_elements = split( /\s+/, $first_header_line1 );
		pop @first_header_line_elements;
		$first_header_id = join( " ", @first_header_line_elements );
	}else{
		$first_header_id = $first_header_line1;
	}

	# second of pair
	if( $second_header_line1 !~ /\S+\s\S+/ ){
		if( $second_header_line1 =~ /(\S*)\/\S*/ ){
			$second_header_id = $1;
		}else{
			$second_header_id = $second_header_line1;
		}
	}elsif( $second_header_line1 =~ /\S+\s\S+/ ){
		my @second_header_line_elements = split( /\s+/, $second_header_line1 );
		pop @second_header_line_elements;
		$second_header_id = join( " ", @second_header_line_elements );
	}else{
		$second_header_id = $second_header_line1;
	}
	if( $first_header_id ne $second_header_id ){
		die "error: header lines in $files[0] and $files[1] do not seem to be paired\n";
	}
}
if( $paired ){
```

```
		if( length($first_sequence_line) >= $length && length($second_sequence_line) >= 
$length ){
			print PAIRED1 $first_header_line1, "\n", $first_sequence_line, "\n", 
$first_header_line2, "\n", $first_quality_line, "\n";
			$count_p1+=1;
			print PAIRED2 $second_header_line1, "\n", $second_sequence_line, "\n", 
$second_header_line2, "\n", $second_quality_line, "\n";
			$count_p2+=1;
		}
		elsif( length($first_sequence_line) < $length && length($second_sequence_line) < 
$length ){
			print DISCARD $first_header_line1, "\n", $first_sequence_line, "\n", 
$first_header_line2, "\n", $first_quality_line, "\n";
			$count_d+=1;
			print DISCARD $second_header_line1, "\n", $second_sequence_line, "\n", 
$second_header_line2, "\n", $second_quality_line, "\n";
			$count_d+=1;
		}
		elsif( length($first_sequence_line) < $length && length($second_sequence_line) >= 
$length ){
			print DISCARD $first_header_line1, "\n", $first_sequence_line, "\n", 
$first_header_line2, "\n", $first_quality_line, "\n";
			$count_d+=1;
			print SINGLE $second_header_line1, "\n", $second_sequence_line, "\n", 
$second_header_line2, "\n", $second_quality_line, "\n";
			$count_s+=1;
		}
		elsif( length($first_sequence_line) >= $length && length($second_sequence_line) < 
$length ){
			print SINGLE $first_header_line1, "\n", $first_sequence_line, "\n", 
$first_header_line2, "\n", $first_quality_line, "\n";
			$count_s+=1;
			print DISCARD $second_header_line1, "\n", $second_sequence_line, "\n", 
$second_header_line2, "\n", $second_quality_line, "\n";
			$count_d+=1;
		}
	}
	else{
		if( length($first_sequence_line) >= $length ){
			print SINGLE $first_header_line1, "\n", $first_sequence_line, "\n", 
$first_header_line2, "\n", $first_quality_line, "\n";
			$count_s+=1;
		}else{
			print DISCARD $first_header_line1, "\n", $first_sequence_line, "\n", 
```

```
$first_header_line2, "\n", $first_quality_line, "\n";
                        $count_d+=1;
                }
        }
}
my @name = split(/\./, $single_file);
my $summaryname= join '.', @name[0..$#name-1];
my $outputname = $summaryname.".summary.txt";
open(FILEOUT, ">$outputname");
if( $paired ){
print FILEOUT "paired1\t$count_p1\n",    "paired2\t$count_p2\n", "single\t$count_s\n",
"discard\t$count_d\n";}
else{
print FILEOUT "single\t$count_s\n", "discard\t$count_d\n";}
close FILEOUT;
close FIRST or die "error: failure closing $files[0]: $!\n";
if( $paired ){
        close SECOND or die "error: failure closing $files[1]: $!\n";
}
close SINGLE or die "error: failure closing $single_file: $!\n";
close DISCARD or die "error: failure closing $discard_file: $!\n";
if( $paired ){
        close PAIRED1 or die "error: failure closing $paired_file1: $!\n";
        close PAIRED2 or die "error: failure closing $paired_file2: $!\n";
}
exit 0 or die "error: $0 ended abnormally: $!\n";
```

Table S1

K17	K19	K21	K23	K25	K27	K29	K31	K33	K35	K37	K39	K41	K43
23825	53912	69572	79045	85509	93356	98891	103680	109728	113506	119195	122100	122904	124573
K45	K47	K49	K51	K53	K55	K57	K59	K61	K63	K65	K67	K69	K71
127947	130691	132755	134728	136704	137585	138763	139603	140464	141555	142021	143541	144530	145309
K73	K75	K77	K79	K81	K83	K85	K87	K89	K91	K93	K95	K97	K99
146636	148466	148758	149472	149703	149042	148051	146459	144175	138962	130525	119665	97355	55904

Supplementary Table 1. The numbers of base pairs of the constructed *Quercus spinosa* cp genome with different assembled k-mers. The longest genome was constructed with k-value of k-mer 81.

Table S2

Total number of reads	Total length of reads (base pair)	Number of contigs	N50 of contigs (base pair)	Minimum contig length (base pair)	Maximum contig length (base pair)
2,781,415	561,845,830	2,121	811	150	7,173

Supplementary Table 2. The assembly results of *Quercus spinosa* chloroplast genome contigs with the most optimal K-mer and the most optimal minimum read length.
